# No Relationship Between Vitamin D Status and Insulin Resistance in a Group of High School Students

**DOI:** 10.4274/jcrpe.507

**Published:** 2011-12-06

**Authors:** Dilek Erdönmez, Şükrü Hatun, Filiz Mine Çizmecioğlu, Alev Keser

**Affiliations:** 1 Kocaeli University of Medical School, Pediatric Endocrinology and Diabetes Unit, Kocaeli, Turkey; +90 262 303 87 31sukruhatun@gmail.comKocaeli University of Medical School, Pediatric Endocrinology and Diabetes Unit, Kocaeli, Turkey

**Keywords:** Vitamin D, metabolic syndrome, insulin resistance

## Abstract

**Objective:** To investigate the effects of vitamin D deficiency on both insulin resistance and risk of metabolic syndrome in children.

**Methods:** The study group consisted of 301 children and adolescents with a mean age of 14.2±1.8 years. Serum 25-hydroxyvitamin D [25(OH)D] levels and insulin resistance indices were evaluated. According to serum 25(OH)D levels, the subjects were classified in 3 groups. Those with levels ≤10 ng/mL were labeled as the vitamin D deficient group (group A), those with levels of 10-20 ng/mL as the vitamin D insufficient group (group B) and those with ≥20 ng/mL as having normal vitamin D levels (group C). Metabolic syndrome was defined according to the International Diabetes Federation consensus. The participants with and without metabolic syndrome were compared in terms of 25(OH)D levels.

**Results:** Mean 25(OH)D level of the total group was 18.2±9.3 (2.8-72.0) ng/mL. Distribution of individuals according to their vitamin D levels showed that 11.6% were in  group A, 53.5% in group B, and 34.9% in group C. The proportions of boys and girls in these categories were  22.9% and 77.1% in group A, 36.6% and 63.4% in group B, 54.3% and 45.7% in group C, respectively. There were no significant differences in 25(OH)D levels in the individuals with and without impaired fasting glucose or impaired glucose tolerance. No relationship was observed between insulin resistance/sensitivity indices and vitamin D status (p>0.05). Metabolic syndrome was diagnosed in 12.3% (n=37) of the children. There was also no difference in mean 25(OH)D levels between individuals who had and those who did not have the metabolic syndrome.

**Conclusion:** In our study, no correlations were found between insulin measurements during oral glucose tolerance test and vitamin D deficiency. Nonetheless, more extended studies including  vitamin D supplementation and evaluating insulin sensitivity via clamp technique are needed to further elucidate this relationship.

**Conflict of interest:**None declared.

## INTRODUCTION

Enhancing the intestinal absorption of calcium, inhibiting PTH gene transcription, regulating the expression of bone matrix proteins and promoting the differentiation of osteoclasts are some of the known effects of vitamin D (1).   Studies on vitamin D receptor (VDR) knock-out mouse models have shown that the immune system is actually normal in these animals, but in the presence of trigger factors, the risk for autoimmune diseases such as type 1 diabetes mellitus and inflammatory bowel diseases increases. These same studies have also found that in the absence of VDRs, there is no increase in the spontaneous occurrence of cancer spontaneously, but a tendency of the mice to develop some tumors that are facilitated by oncogenes and chemocarcinogens has been observed. Additionally, a predisposition to high-renin hypertension, cardiac hypertrophy and thrombosis is seen in the absence of these receptors (2). In recent years, there is a growing interest in the non-classical effects of vitamin D, which is based on findings showing the presence of VDRs in tissues other than bone, gut and kidneys (3). There are studies in adults  reporting that vitamin D deficiency affects insulin sensitivity negatively and increases the risk for type 2 diabetes mellitus (4,5,6,7). There are also some reports showing a correlation (mild or very strong) of vitamin D deficiency with metabolic syndrome and insulin sensitivity in children (8,9). In this present study, the effects of vitamin D deficiency on both insulin sensitivity and risk of metabolic syndrome were investigated in a region which is known to have a high incidence of vitamin D deficiency among the adolescents (10). 

## MATERIALS AND METHODS

Serum 25-hydroxyvitamin D [25(OH)D] levels and oral glucose tolerance test (OGTT) results of 301 primary and high school students [177 girls (59%) and 124 boys (41%)] were evaluated at the end of the winter season. The mean age of the subjects was 14.2±1.8 (range: 11.0-18.7) years. Weight and height of each child were measured according to standard methods. The study group was also assessed for obesity and metabolic syndrome. 

All cases underwent an OGTT after an 8-12-hour overnight fast. Insulin resistance and sensitivity indices were calculated in all children, using the following equations: fasting glucose/insulin ratio (FGIR) (fasting glucose/fasting insulin), homeostasis model for assessment of insulin resistance (HOMA-IR) [fasting glucose (nmol/L) x fasting insulin (mIU/mL)/22.5] and quantitative insulin sensitivity check index (QUICKI) [1/log insulin + log glucose (mg/L)]. 

According to the American Diabetes Association (ADA) recommendations, impaired fasting glucose (IFG) is defined as a fasting glucose of ≥100 mg/dL and impaired glucose tolerance (IGT) is defined as a two-hour glucose level of ≥140 mg/dL. 

A competitive protein binding assay was used to measure 25(OH)D levels in fasting serum samples (Vit D EIA kit, Immundiagnostic, Bensheim, Germany). The normal range for 25(OH)D in this assay was set at 11-70 ng/mL and intra-and interassay coefficients of variation (CVs) were 10.7% and 13.2%, respectively. Serum 25(OH)D levels were categorized as follows: ≤10 ng/mL indicating vitamin D deficiency (group A), 10-20 ng/mL indicating vitamin D insufficiency (group B), and ≥20 ng/mL for normal vitamin D level (group C). 

Metabolic syndrome was defined according to the International Diabetes Federation (IDF) consensus. The relationship between vitamin D status (insufficiency, deficiency and normal) and insulin resistance indices was evaluated. The participants with and without metabolic syndrome were compared in terms of 25(OH)D levels. Body mass index (BMI) of each child was calculated as weight (kg)/height (m)2.

The data were analyzed with the Statistical Package for the Social Sciences (SPSS Inc., Chicago), Version 11.5. A two-sample (independent group) t-test was performed to compare some characteristics and 25(OH)D categories between boys and girls. The chi-square test was used to assess the frequency differences between 25(OH)D categories. The relationships between 25(OH)D and insulin resistance indices were evaluated using the Pearson’s correlation coefficient. 

A p-value of <0.05 was accepted to be statistically significant. Data are presented as mean±SD values.

## RESULTS

Mean BMI was 26.0±0.0 (19.3-40.3), being higher in the girls (26.5±3.8, range: 19.9-37.7) than in the boys (25.2±3.7, range: 19.3-40.3) (p=0.002). Mean 25(OH)D level of the total group was 18.2±9.3 ng/mL (2.8-72.0) [20.7±9.5 ng/mL (2.8-72) in the boys and 16.4±8.8 ng/mL (6.8-72) in the girls]. 25(OH)D levels were lower in the girls than in the boys (p<0.001). Distribution of individuals according to their 25(OH)D levels showed that 11.6% were in  group A, 53.5% in group B and 34.9% in group C. The proportions of boys and girls in these categories were 22.9% and 77.1% in group A, 36.6% and 63.4% in group B, and 54.3% and 45.7% in group C, respectively. Between groups also, vitamin D deficiency was higher in the girls (χ2=13.662; p=0.00). There was no relationship between degree of vitamin D deficiency and obesity (χ2=0.596; p>0.05). IFG and IGT rates were computed as 8% (n=24) and 5% (n=15), respectively. 

There were no significant differences in 25(OH)D levels between subjects who had and those who did not have IFG or IGT. Frequency of IFG was 20.8% in group A (n=5), 45.8% in group B (n=11), and 33.8% in group C (n=8) (χ2=2.204; p>0.05). IGT frequency in group A was 6.7% (n=1), in group B - 40% (n=6), and in group C was 53.3% (n=8) (χ2=2.412; p>0.05). No significant associations were found between insulin resistance/sensitivity indices and vitamin D status (p>0.05). These results are summarized in Tables 1 and 2. Frequency of metabolic syndrome was found to be 12.3% (n=37). The mean vitamin D levels were similar in subjects with and without metabolic syndrome [20.0±13.7 ng/mL (6.6±70) vs. 17.8±8.5 ng/mL (2.8±72)] (χ2=0.419; p>0.05)

**Tables 1:**
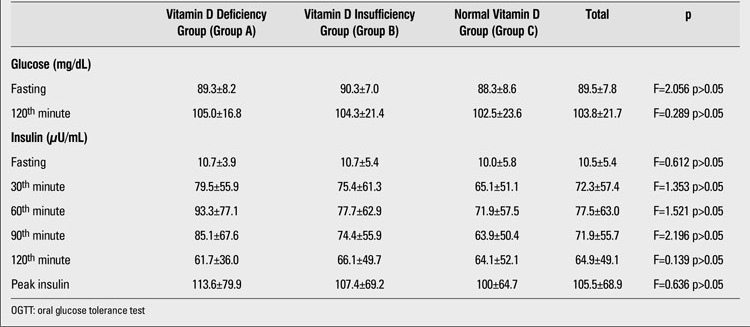
Glucose and insulin levels obtained during OGTT in children with vitamin D deficiency, vitamin D insufficiency and normal vitamin D levels

**2:**
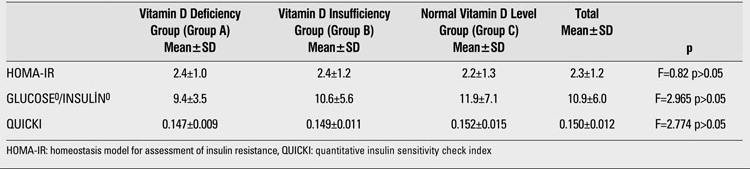
Relationships between insulin resistance indices and vitamin D status

## DISCUSSION

The role of inflammatory cytokines in the relationship between obesity and insulin resistance is known and it is emphasised that vitamin D modulates the production of cytokines (11,12,13). It has been suggested that vitamin D insufficiency decreases insulin sensitivity in this way and also increases the risk of type 2 diabetes (14,15). However, these associations, as well as the relationships between severity and/or duration of vitamin D deficiency and insulin sensitivity, are issues which are not clear and which require further documentation (16). 

Frequency of obesity and metabolic syndrome is on the increase among school children in Turkey (17). In addition, vitamin D insufficiency and deficiency have been reported in 86.5% of school children in our region (10). In this study, we found no association of vitamin D level with insulin resistance, impaired glucose balance and metabolic syndrome. 

The results of the 2001-2004 National Nutrition and Health Survey in the United States indicate that metabolic syndrome prevalence was 3.8 fold higher among obese adolescents whose 25(OH)D levels were lower than 15 ng/mL as compared to those with levels higher than 26 ng/mL (8). The results of this same survey also show, independently of adiposity, a strong association between low vitamin D level and metabolic syndrome as well as an association of low vitamin D level with hypertension and hyperglycaemia. On the other hand, another study conducted among adolescents of French origin in Canada failed to reveal an association between 25(OH)D level and existence of at least two components of metabolic syndrome (9). In this same study, it was shown that every 10 ng/mL increment in 25(OH)D level causes a mild decrease in the fasting blood glucose levels and HOMA-IR. As is well known, discussions and controversies about both metabolic syndrome diagnostic criteria and evaluation of insulin resistance continue to this day. Many researchers agree that the calculations based on fasting blood glucose level are inadequate to evaluate the whole body insulin resistance (18). It must be taken into account that in the aforementioned studies, insulin resistance was evaluated according to fasting blood glucose levels. The golden diagnostic method for insulin sensitivity is euglycemic hyperinsulinemic clamp. However, insulin levels obtained during OGTT were reported to correlate strongly with clamp results (18,19,20). 

In conclusion, this study failed to show any relationship between insulin sensitivity and vitamin D status. Nonetheless, more extended studies also including  vitamin D supplementation and evaluating insulin sensitivity via clamp technique are needed to further elucidate this topic.

## References

[1] Demay MB, Sabbagh Y, Carpenter TO (2007). Calcium and vitamin D: What is known about the effects on growing bone. Pediatrics.

[2] Bouillon R, Carmeliet G, Verlinden L, van Etten E, Verstuyf A, Luderer HF, Lieben L, Mathieu C, Demay M (2008). Vitamin D and human health: lessons from vitamin D receptor null mice. Endocr Rev.

[3] Bikle D (2009). Nonclassic actions of vitamin D. J Clin Endocrinol Metab.

[4] Gulseth HL, Gjelstad IM, Tierney AC, Lovegrove JA, Defoort C, Blak EE, Lo ez  Miranda J, Kiec Wilk B, Ris U, Roche HM, Drevon CA, Birkeland KI (2010). Serum vitamin D concentration does not predict insulin action or secretion in European subjects with the metabolic syndrome. Diabetes Care.

[5] Chiu KC, Chu A, Go VL, Saad MF (2004). Hypovitaminosis D is associated with insulin resistance and beta cell dysfunction. Am J Clin Nutr.

[6] Zhao G, Ford ES, Li C (2010). Associations of serum concentrations of 25-hydroxyvitamin D and parathyroid hormone with surrogate markers of insulin resistance among U.S. adults without physician-diagnosed diabetes: NHANES, 2003-2006. Diabetes Care.

[7] Kayaniyil S, Vieth R, Retnakaran R, Knight JA, Qi Y, Gerstein HC, Perkins BA, Harris SB, Zinman B, Hanley AJ (2010). Association of vitamin D with insulin resistance and beta-cell dysfunction in subjects at risk for type 2 diabetes. Diabetes Care.

[8] Reis JP, von Mühlen D, Miller ER 3rd, Michos ED, Appel LJ (2009). Vitamin D status and cardiometabolic risk factors in the United States adolescent population. Pediatrics.

[9] Delvin EE, Lambert M, Levy E, O'Loughlin J, Mark S, Gray-Donald K, Paradis G (2010). Vitamin D status is modestly associated with glycemia and indicators of lipid metabolism in French-Canadian children and adolescents. J Nutr.

[10] Hatun S, Islam O, Cizmecioglu F, Kara B, Babaoglu K, Berk F, Gokalp AS (2005). Subclinical vitamin d deficiency is increased in adolescent girls who wear concealing clothing. J Nutr.

[11] Boonstra A, Barrat FJ, Crain C, Heath VL, Savelkoul HF, O'Garra A (2001). 1 alpha, 25-dihydroxyvitamin D3 has a direct effect on naive CD4(1) T cells to enhance the development of Th2 cells. J Immunol.

[12] Willheim M, Thien R, Schrattbauer K, Bajna E, Holub M, Gruber R, Baier K, Pietschmann P, Reinisch W, Scheiner O, Peterlik M (1999). Regulatory effects of 1 alpha, 25 dihydroxyvitamin D3 on cytokine production of human peripheral blood lymphocytes. J Clin Endocrinol Metab.

[13] Rigby WF, Denome S, Fanger MW (1987). Regulation of lymphokine production and human T-lymphocyte activation by 1,25 dihydroxyvitmin D3: Specific inhibition at the level of messenger RNA. J Clin Invest.

[14] Mathieu C, Gysemans C, Giulietti A, Bouillon R (2005). Vitamin D and diabetes.. Diabetologia.

[15] Holick MF (2008). Diabetes and the vitamin d connection. Curr Diab Rep.

[16] Binkley N, Ramamurthy R, Krueger D (2010). Low vitamin D status: definition, prevalence, consequences, and correction. Endocrinol Metab Clin North Am.

[17] Cizmecioglu FM, Etiler N, Hamzaoglu O, Hatun S (2009). Prevalence of metabolic syndrome in schoolchildren and adolescents in Turkey: a population-based study. J Pediatr Endocrinol Metab.

[18] Levy-Marchal C, Arslanian S, Cutfield W, Sinaiko A, Druet C, Marcovecchio ML, Chiarelli F; ESPE-LWPES-ISPAD-APPES-APEG-SLEP-JSPE; Insulin Resistance in Children Consensus Conference Group (2010). Insulin resistance in children: consensus, perspective, and future directions. J Clin Endocrinol Metab.

[19] Stumvoll M, Mitrakou A, Pimenta W, Jenssen T, Yki-Ja¨rvinen H, Van Haeften T, Renn W, Gerich J (2000). Use of the oral glucose tolerance test to assess insulin release and insulin sensitivity. Diabetes Care.

[20] Yeckel CW, Weiss R, Dziura J, Taksali SE, Dufour S, Burgert TS, Tamborlane WV, Caprio S (2004). Validation of insulin sensitivityindices from oral glucose tolerance test parameters in obese children and adolescents. J Clin Endocrinol Metab.

